# Isolation, Cytotoxicity Evaluation and HPLC-Quantification of the Chemical Constituents from *Prangos pabularia*

**DOI:** 10.1371/journal.pone.0108713

**Published:** 2014-10-14

**Authors:** Saleem Farooq, Shakeel-u-Rehman, Nisar Ahmad Dangroo, Dev Priya, Javid Ahmad Banday, Pyare Lal Sangwan, Mushtaq Ahmad Qurishi, Surrinder Koul, Ajit Kumar Saxena

**Affiliations:** 1 Bio-organic Chemistry Section, CSIR-Indian Institute of Integrative Medicine, Jammu, India; 2 Bio-organic Chemistry Section, CSIR-Indian Institute of Integrative Medicine, Srinagar, India; 3 Department of Chemistry, University of Kashmir, Srinagar, India; 4 Cancer Pharmacology Division, CSIR-Indian Institute of Integrative Medicine, Jammu, India; University of Vigo, Spain

## Abstract

Phytochemical analysis of the dichloromethane:methanol (1∶1) extract of root parts of *Prangos pabularia* led to the isolation of twelve cytotoxic constituents, viz., 6-hydroxycoumarin (**1**), 7-hydroxycoumarin (**2**), heraclenol-glycoside (**3**), xanthotoxol (**4**), heraclenol (**5**), oxypeucedanin hydrate (**6**), 8-((3,3-dimethyloxiran-2-yl)methyl)-7-methoxy-2H-chromen-2-one (**7**), oxypeucedanin hydrate monoacetate (**8**), xanthotoxin (**9**), 4-((2-hydroxy-3-methylbut-3-en-1-yl)oxy)-7H-furo[3,2-g]chromen-7-one (**10**), imperatorin (**11**) and osthol (**12**). The isolates were identified using spectral techniques in the light of literature. 3-(4,5-dimethyl thiazol-2yl)-2,5-diphenyltetrazolium bromide (MTT) cytotoxicity screening of the isolated constituents was carried out against six human cancer cell lines including lung (A549 and NCI-H322), epidermoid carcinoma (A431), melanoma (A375), prostate (PC-3) and Colon (HCT-116) cell lines. Osthol (**12**) exhibited the highest cytotoxicity with IC_50_ values of 3.2, 6.2, 10.9, 14.5, 24.8, and 30.2 µM against epidermoid carcinoma (A431), melanoma (A375), lung (NCI-H322), lung (A549), prostate (PC-3) and colon (HCT-116) cell lines respectively. Epidermoid carcinoma cell line A431 was sensitive to most of the compounds followed by lung (A549) cancer cell line. Finally a simple and reliable HPLC method was developed (RP-HPLC-DAD) and validated for the simultaneous quantification of these cytotoxic constituents in *Prangos pabularia*. The extract was analyzed using a reversed-phase Agilent ZORBAX eclipse plus column C_18_ (4.6×250 mm, 5 µm) at 250 nm wavelength using a gradient water-methanol solvent system at a flow rate of 0.8 ml/min. The RP-HPLC method is validated in terms of recovery, linearity, accuracy and precision (intra and inter-day validation). This method, because of shorter analysis time, makes it valuable for the commercial quality control of *Prangos pabularia* extracts and its future pharmaceutical preparations.

## Introduction

*Prangos*, one of the largest and most widely distributed genus of family Umbelliferae consists of around 30–40 species. Most of the *Prangos* species are reported to possess diverse pharmacological activities viz. anti-microbial [1], anti-oxidant [2], cytotoxic [3], [4], anti-helmintic and aphrodisiac [5], besides, their use in the treatment of haemorrhoids, wounds and leukoplakia [6]. In Central Asia, the extracts of *Prangos* species have been used to stop bleeding and heal scars [4].

*Prangos pabularia* commonly known as “Komal” in Hindi and “Kurangas” locally (Kashmir), occurs in stony slopes of Ladakh (Jammu and Kashmir, India). It is the only species of the genus found in India. In Indian traditional system of medicine, its roots and fruits have been used as diuretic, carmative, laxative, stimulant and liver tonic [7]. It is also used for treatment of itches, and as a promoter for the expulsion of foetus [7]. An infusion of the roots is useful in indigestion, flatulence and regularization of menstrual cycle in females [8].

Previous phytochemical investigations on the fruits and roots of *p. pabularia* revealed the presence of various chemical constituents, consisting of coumarins of diversified structures, terpenoids and glycosides [9]–[11]. It may be pertinent to say that coumarins form an important class of compounds known to possess various pharmacological activities viz. anti-inflammatory, anti-pyretic [12], anti-oxidant [13], bronchodilator [14], vasodilator [15], anti-amoebic [16], anti-bacterial [17] and anti-fungal [18]. Physiological, bacteriostatic and antitumor activity make these compounds attractive for further backbone derivatization and screening as novel therapeutic agent/s [19]. Weber and co-workers have shown that coumarin and its metabolite 7-hydroxycoumarin exhibit antitumor activity against several human tumour cell lines. In addition, it has been shown that 4-hydroxycoumarin and 7-hydroxycoumarin inhibit cell proliferation in a gastric carcinoma [20]. Recently, our research group demonstrated the cytotoxic activity of a novel library of 6-Hydroxycoumarin linked triazole and isoxazole derivatives as potent and selective cytotoxic agents against prostate (PC-3) and lung (A-549) cancer cell lines [21]. Osthol, one of the major constituents of the plant has been found to be a potent respiratory and circulatory stimulant in experimental animals [22], [23]. Osthol analogs have been found to possess exceptional pharmacological activities, e.g., insecticidal activity [24], anti-microbiology [25], anti-inflammatory [26] and anti-cancer [27]. Osthol has been reported to suppress the migration and invasion of A549 human lung cancer cells through inhibition of matrix metalloproteinase-2 and matrix metalloproteinase-9 *in vitro* [28]. In addition to this, osthol has also been reported to induce apoptosis and inhibit proliferation of human osteosarcoma cells [29]. Recently, our research group reported the broad-spectrum cytotoxic activity of a novel library of triazolyl derivatives of osthol against various cancer cell lines. These triazolyl derivatives are believed to induce apoptosis in Colo-205 cell line through the disruption of mitochondrial membrane potential (ΛΨm) [30].

Previously Koul *et al*., (1978, 1979) reported novel furanocoumarin glycosides and the essential oil composition of this plant species [31], [32]. The chemical transformation studies on the isolated coumarins are also reported. The thermal studies of one its constituents oxypeucedanin hydrate monoacetate crystal, has been studied for its possible use as a candidate for modern optoelectronic devices [33].

We herein report the complete secondary metabolite constitution of *Prangos pabularia*. The isolates were evaluated using 3-(4,5-dimethyl thiazol-2yl)-2,5-diphenyltetrazolium bromide (MTT) cytotoxicity assay against human lung (A549 and NCI-H322), epidermoid carcinoma (A431), melanoma (A375), prostate (PC-3) and colon (HCT-116) cancer cell lines. Osthol (**12**) proved to be the most potent bioactive constituent against epidermoid carcinoma (A431) and melanoma (A375) cell lines. Finally a chemo biological HPLC standardisation method was developed to simultaneously quantify the isolated constituents of *Prangos pabularia*. An exhaustive literature survey revealed that quantification of single component (bergapten) from this plant has been recently reported [34], however this study forms the first report with regard to isolation, cytotoxic studies and simultaneous separation and quantification of the constituents from the aforementioned plant.

## Materials and Methods

### 1. General experimental procedures

Following instruments were used to carry out the physical and spectral data: IR spectra on Perkin Elemer FT-IR spectrometer as KBr Pellet or neat sample. NMR spectra on 500, 400 and 125 MHz Bruker spectrometers in CDCl_3_ with TMS as internal standard, chemical shift is expressed in *δ* (ppm) and coupling constant in Hertz. Mass analysis was carried out using Nexera UHPLC @ 130 Mpa with SIL-30 AC Nexera autosampler coupled to an LC–MS 8030 tandem mass spectrometer manufactured by Shimadzu Corporation, Kyoto, Japan. All the compounds were analysed in full scan mode with nitrogen servin gas an interface gas. Detection was done in + APCI mode having probe voltage of 180.0 V, with probe temperature of 400°C and nebulising gas flow of 2.5 L/min. Column chromatography was carried on normal phase silica gel 60–120 mesh (Merck grade), precoated TLC plates with silica gel 60 F254(Merck, 0.25 mm). Detection was done by using UV torch, iodine vapours and cerric sulphate.

### 2. Plant material collection

The plant material of *P. pabularia* was collected from stony slopes of Ladakh (Drass, J&K, India) in July 2008 prior to proper permission from the Department of Forestry (J&K, India). The plant material was identified and authenticated by Dr. Akhtar H. Malik, (University of Kashmir). The specimen has been deposited under accession No. 33214 and Collection No. 1203- Javid, Kash. This plant does not fall in the list of endangered species, therefore, there are no specific concerns of its extinction.

### 3. Preparation of extract

The root parts of the plant were processed separately, chopped, oven dried at 40°C and ground to a fine powder. The powdered root (2.0 kg) was sequentially extracted using DCM:Methanol (1∶1) solvent system. The solvent was removed under vacuo on a rotary evaporator to afford crude DCM:Methanol (1∶1) extract of root (120.0 g).

### 4. Isolation of the chemical constituents

30.0 g of the DCM:Methanol (1∶1) extract of root thus obtained was subjected to column chromatography and eluted with solvents of increasing polarity and the fractions collected thereof were subjected to repeated column chromatography and re-crystallization techniques yielding twelve compounds. The purified compounds (>98% purity) were characterised by spectral data analysis (^1^H NMR, ^13^C NMR, HRMS, and IR spectroscopy) in the light of literature [35]–[41].

### 5. Evaluation of bioactivity of the extracts and isolated constituents using MTT cytotoxicity assay

The cytotoxic effect of DCM:Methanol (1∶1) extract of root as well as the isolates was evaluated using MTT assay. All the human cancer cell lines [lung (A549 and NCI-H322), epidermoid carcinoma (A431), melanoma (A375), prostate (PC-3) and colon (HCT-116)] were obtained from ATCC via Sigma/Aldrich St. Louis, Mo, USA. Cells used were grown in RPMI-1640 medium containing 10% foetal bovine serum (FBS), 100 unit penicillin/100 µg Streptomycin per ml medium. Cells were allowed to grow in carbon dioxide incubator (Thermo scientific USA) at 37°C with 98% humidity and 5% CO_2_ gas environment in case of MTT assay. In the present case, all cell lines seeded in flat-bottomed 96-well plates were allowed to adhere overnight, and then media containing different samples (varying concentrations) were added. Cell viability of the compounds treated cells was measured by using MTT assay. Briefly, cells (10^4^ cells/well) were cultured in 96 well tissue culture plates and treated with different concentrations of compounds for 48 h. At the end of incubation, 20 µL of MTT (2.5 mg/ml) was added to the wells and incubated for 4 h. Absorbance was recorded at 570 nm using Eliza Plate Reader.

### 6. RP-HPLC analysis

#### 6.1. Preparation of sample solutions

Stock solutions of 1.0 mg/ml of isolated constituents were prepared and appropriate dilutions made for HPLC analysis. 10.4 mg of DCM:Methanol (1∶1) extract of *P. pabularia* was dissolved separately in 2.4 ml of solvent and simultaneously diluted to obtain various concentrations. The solutions were filtered through 0.45 µm PTFE filter prior to HPLC analysis. The solutions were stored at −20.0°C when not in use and they were stable for at least six months.

#### 6.2. HPLC instrument and chromatographic conditions

Quantitative HPLC analysis was performed on Agilent 1200 series HPLC system equipped with a HPLC quaternary pump (G13311A), auto sampler (G1329A), degasser (G1322A), UV detector (G1315D) and column oven (G1316B) controlled by EZchrome software which was used for data analysis and processing. Separation was carried out on RP-C18 column (4.6×250 mm; particle size5 µm; Agilent, Zorbax Eclipse plus) with column oven temperature of 25°C using a gradient elution of eluent B (water) and C (methanol) was used for the separation of target analytes. The gradient programme was as follows: 0–5 min, 20% C; 5–10 min, 50% C; 10–15 min, 70% C; 15–18 min 80% C; 18–20 min, 90% C; 20–25 min, 95% C; and 25–30 min, 20% C. Solvent flow rate of 0.8 ml/min was maintained throughout the analysis and the injection volumes ranging from 2.0 to 10.0 µl. All the analysis was carried out at room temperature at a wavelength of 250 nm with run time 30 min.

### 7. Validation of the method

The HPLC method developed for *Prangos pabularia* was validated for specificity, linearity, accuracy, precision and quantification parameters, viz., LOD and LOQ.

#### 7.1. Specificity

To validate the specificity of the developed method standard solutions of the analytes and that of the extract of *Prangos pabularia* were prepared. A gradient system of water and methanol was used as a blank control. A fixed injection volume each of sample, standard and blank solutions was given and analysed using developed HPLC method.

#### 7.2. Linearity

In order to validate the linearity of the developed method solutions of marker compounds (0.833–83.33 µg/ml) were prepared. A 10.0 µL volume of standard solution was injected and analysed using the HPLC method as described above. The analysis was done in triplicate for all the samples. Calibration curves for all the compounds were obtained using linear regression analysis (see Data S1).

#### 7.3. Precision

The standard solutions containing 0.833–83.33 µg/ml corresponding to test ranges of twelve marker compounds were prepared from stock solutions and calibration curves were constructed and their linear ranges determined. Calibration curves were plotted by the peak area versus concentration of each analyte. The linearity was evaluated by linear regression equation (y = mx+c) calculated by the least square regression method. All calibration curves showed good linearity regressions under the current chromatographic conditions. The limit of detection (LOD) and limit of quantification (LOQ) were calculated using the equations: LOD = 3.3 σ/S and LOQ = 10 σ/s.

Where σ and S represent the standard deviation of response and the slope of the calibration curve respectively.

Limit of detection represents the lowest concentration of the analyte in a sample that can be detected by the HPLC under the developed method while as limit of quantification represents the lowest concentration that can be quantified with acceptable precision and accuracy under the operating conditions.

Recovery studies were carried to determine accuracy of the developed assay. The known amount of investigated compound was added to the accurately weighed portion of plant extract, processed and analysed. The recovery percentage was calculated by the formula [34].

Recovery (%)  =  (amount found-amount present)/amount spiked ×100.

Stability of the compounds was examined by analysing the standard solution at 0, 2, 4, 12, 24 and 48 hrs after storage at room temperature for two days. The samples were also analysed after storage for 3 months at 4°C and even after a storage of 6 months at −20°C, the results obtained indicated that the solutions were stable for a at least 6 months.

#### 7.4. Quantification of the constituents

Using the developed method the concentration of constituents was determined on dry weight basis of plant material.

#### 7.5 Statistical analysis

Each experiment was done in triplicate and mean values were calculated. The data were recorded as means ± standard deviations. Analysis of variance for individual parameters was performed on the basis of mean values to find out the significance at p<0.05.

## Results and Discussion

### 1. Phytochemistry

**Figure 1:**
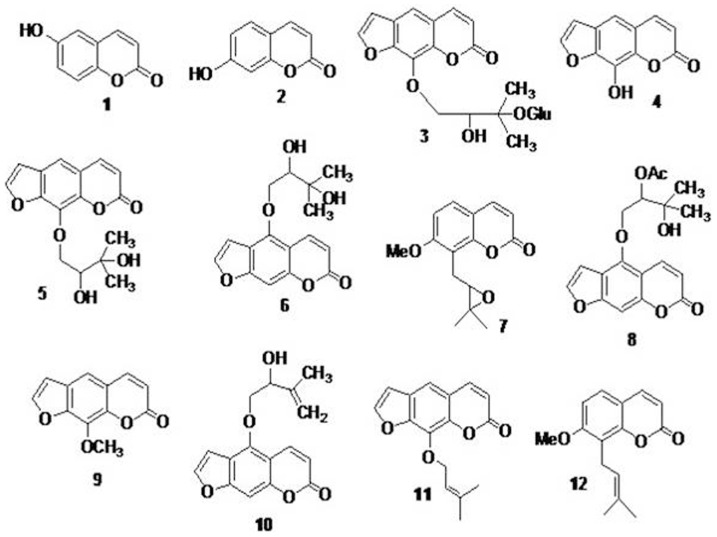
Chemical structure of marker compounds isolated from plant Prangos pabularia. (**1**), 6-hydroxo-coumarin (**2**), Umbelliferone (**3**), Heraclenol glycoside (**4**), Xanthotoxol (**5**), Heraclenol (**6**), Oxypeucedanin hydrate (**7**), 8-((3,3-dimethyloxiran-2-yl)methyl)-7-methoxy-2H-chromen-2-one (Merangin) (**8**), Oxypeucedanin hydrate monoacetate (**9**), Xanthotoxin (**10**), 4-((2-hydroxy-3-methylbut-3-en-1-yl)oxy)-7H-furo[3,2-g]chromen-7-one (**11**), Imperatorin (**12**), Osthol.

Phytochemical investigation of DCM:methanol (1∶1) extract yielded twelve compounds, viz., 6-hydroxycoumarin (**1**), 7-hydroxycoumarin (**2**), heraclenol-glycoside (**3**), xanthotoxol (**4**), heraclenol (**5**), oxypeucedanin hydrate (**6**), 8-((3,3-dimethyloxiran-2-yl)methyl)-7-methoxy-2H-chromen-2-one (**7**), oxypeucedanin hydrate monoacetate (**8**), xanthotoxin (**9**), 4-((2-hydroxy-3-methylbut-3-en-1-yl)oxy)-7H-furo[3,2-g]chromen-7-one (**10**), imperatorin (**11**) and osthol (**12**). Structures of all the compounds are depicted in (Fig. 1) and were characterised using spectral data in the light of literature [31]–[37]. The biological studies of osthol and related molecules containing coumarin ring, carried out in last few years have provided an additional dimension to the bioactivity profile of the title compound osthol. The potential of osthol and other coumarins has not been fully exploited despite its biological importance; therefore, more efforts towards the building of diverse libraries around its chemical structure and their biological profile are in demand. Thus keeping in view the above said merits of natural product isolation and their synthetic modification for the development of drug like molecules, we were interested in the development of standard HPLC methods (resolution and quantification) of the natural product isolates of *P. Pabularia*.

### 2. Biological evaluation

MTT cytotoxicity assay was used to screen the isolated constituents against a panel of six human cancer cell lines i.e., human lung (A549 and NCI-H322), epidermoid carcinoma (A431), melanoma (A375), prostate (PC-3) and colon (HCT-116). Most of the isolated compounds displayed broad spectrum cytotoxic effect in a dose dependent manner. The compounds which exhibited significant cytotoxic effect, greater than 50% growth inhibition at the preliminary screening concentration (50 µM) were assayed using MTT assay at different concentrations (2.0–50.0 µM) to generate the IC_50_ values (Table 1). BEZ-235 was used as positive control in this assay. The values are the average of triplicate analysis. Almost all the compounds displayed significant cytotoxity against the tested cancer cell lines.

**Table 1:** Cytotoxicity profile of the isolated constituents using MTT cytotoxicity assay.

Cell line	A549	A431	NCI-H322	PC-3	A375	HCT-116
Tissue type	Lung	Epidermoid carcinoma	Lung	Prostate	Melanoma	Colon
Compound	**IC_50_ in (µM)**					
**1**	>50	41±1.12	>50	>50	>50	37.0±1.88
**2**	>50	11.3±0.88	>50	>50	>50	>50
**3**	>50	12.9±0.56	6.9±0.42	>50	25.6±1.08	>50
**4**	23.3±1.58	>50	25.8±1.27	37.3±1.54	>50	>50
**5**	47.5±2.02	4.6±0.22	13.2±0.46	24.8±0.98	>50	>50
**6**	>50	4.8±0.25	23.8±1.10	>50	>50	>50
**7**	21.7±1.18	28.5±1.10	32.0±1.12	>50	32.8±1.83	34.5±1.73
**8**	>50	16.0±0.91	34.8±1.28	34.4±1.75	22.4±0.84	>50
**9**	>50	37.8±0.98	46.4±2.20	46.8±2.19	44.0±2.02	>50
**10**	22.0±1.26	14.0±0.82	31.0±1.82	18.0±0.62	18.0±0.42	>50
**11**	16.6±1.20	25.3±1.21	23.3±1.54	>50	11.0±0.49	41.0±2.27
**12**	14.5±0.95	3.2±0.20	10.9±0.73	24.8±1.23	6.2±0.28	30.2±1.95
**BEZ-235**	6.50	12.50	10.30	12.30	9.32	0.044

**IC_50_** values are indicated as the mean ± SD of three independent experiments.

**BEZ-235** was used as positive control.

Osthol (**12**), a highly active molecule displayed a broad spectrum cytotoxic profile against all the tested cancer cell lines, viz., lung (A549 and NCI-H322), epidermoid carcinoma (A431), melanoma (A375), prostate (PC-3) and Colon (HCT-116) with corresponding IC_50_ values of 3.2, 6.2, 10.9, 14.5, 24.8, and 30.2 µM respectively. Osthol (**12**) exhibited superior potency to BEZ-235 against A431 and A375 cells. 7-Hydroxycoumarin (**2**), heraclanol (**5**) and oxypeucedanin hydrate (**6**) also displayed better cytotoxicity than BEZ-235 against A431 cell line with IC_50_ of 11.3, 4.6 and 4.8 µM respectively. Heraclanol-glycoside (**3**) displayed slightly better activity than BEZ-235 against NCI-H322 cancer cell line with IC_50_ of 6.9 µM while as it was weakly active towards the other cancer cell lines.

From the results, it may be summarized that the compounds in general were more specific to epidermoid carcinoma (A431), followed by lung cell line (NCI-H322). It would be of interest to investigate the anticancer profiles of the compounds after incurring modification in some of these active molecules such as **5**, **6** and **12** so as to develop more potent and less toxic compounds than the parent ones.

### 3. HPLC method development

Adequate resolution is necessary to acquire satisfactory quantification of compounds. In order to obtain chromatograms with baseline separation of the twelve marker compounds in *P. Pabularia*, selection of the column, mobile phase composition, conditions for gradient flow and temperature was performed by HPLC. Initially a number of solvent systems were tried to develop a separation method for the isolates from *P. pabularia* extract solutions starting from pure methanol and constantly adding the aqueous-phase. Methanol and water solvent system proved to be most effective and therefore, selected for the present study. Finally a simple gradient method comprising of B (water) and C (methanol) as: 0–5 min, 20% C; 5–10 min, 50% C; 10–15 min, 70% C; 15–18 min 80% C; 18–20 min, 90% C; 20–25 min, 95% C; and 25–30 min, 20% C at a flow rate of 0.8 ml/min provided a best base line separation with elution of all 12 marker compounds in less than 30 minutes. Maximally efficient detection was observed at a fixed wavelength of 250 nm.

**Figure 2:**
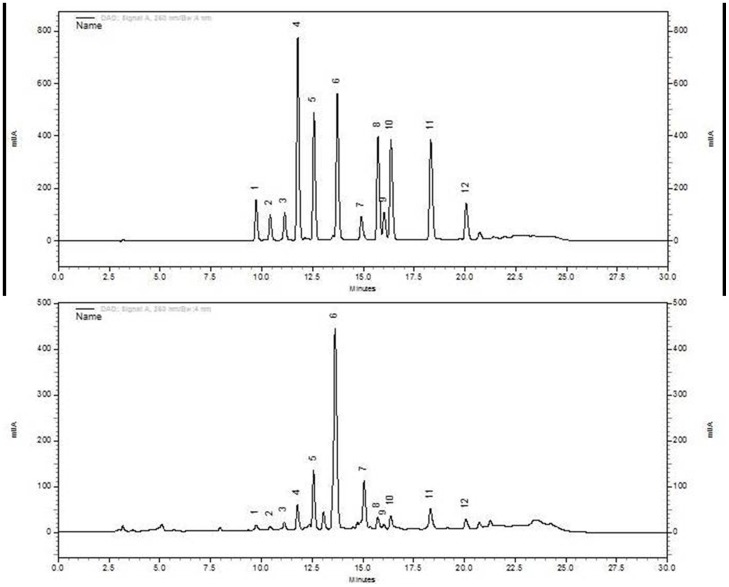
(a) HPLC chromatogram of the standards isolated from *P. pabularia* and (b) HPLC chromatogram of the DCM: Methanol (1∶1) extract solution of *P. pabularia*.

Compounds **1**, **2**, **3**, **4**, **5**, **6**, **7**, **8**, **9**, **10**, **11** and **12** were found to elute at 9.66, 10.27, 11.07, 11.54, 12.43, 13.55, 14.80, 15.49, 15.81, 16.13, 18.10 and 19.90 min respectively with highly symmetric and well resolved peaks. The HPLC chromatograms are shown in (Fig. 2) which depicts a good separation of the peaks for all the analytes tested.

#### 3.1. Specificity of developed method

The developed method depicted a fair amount of specificity. Comparison between the peaks of the spectra and the retention times ascertained the specificity of the developed analytical methods. Assessment of the spectral peaks at three positions, viz., start, apex and end was observed with high degree of symmetry with well resolved chromatogram as shown in (Fig. 2). As it can be seen, no interference of peaks is observed in the HPLC chromatogram which establishes high sensitivity of the developed method.

#### 3.2. Linearity

Linearity of the developed method was validated by analysing ten concentrations of each analyte ranging between 0.83 and 83.33 µg/ml. The concentration range is generally chosen as per ICH guidelines (International Conference on Harmonization i.e., 70% and 130% of the nominal concentration). Thus in a common way we prepared only one stock solution and subsequently diluted it to the different concentration levels both above and below the nominal concentrations for each analyte. Triplicate analysis for each analyte was carried out. Good correlation coefficients ranging between 0.9992 and 0.9999 were observed (Data S1). The standard calibration curves were all linear in the tested ranges. Other quantification parameters such as LOD and LOQ have also been calculated. LOD and LOQ of these cytotoxic constituents ranged between 0.012 to 0.382 and 0.042 to 1.090 µg/ml respectively indicating that the developed method for *P. pabularia* exhibited good sensitivity. All the results are depicted in (Table 2).

**Table 2:** Limits of detection (LOD) and quantification (LOQ) of marker compounds **1–12**.

Marker compound	Calibration curve ^a)^	R^2^	Linear range(µg/mL)	LOD^b)^ (µg/mL)	LOQ^c)^ (µg/mL)
1	y = 3.31618e-005x - 0.117756	0.9999	0.83–83.33	0.065	0.174
2	y = 4.92386e-005x - 0.464667	0.9998	0.83–83.33	0.094	0.261
3	y = 3.31618e-005x - 0.117756	0.9999	0.83–83.33	0.073	0.251
4	y = 6.32890e-006x - 0.237669	0.9999	0.83–83.33	0.012	0.042
5	y = 1.00886e-005x - 0.121333	0.9999	0.83–83.33	0.020	0.073
6	y = 8.43083e-006x - 0.619976	0.9997	0.83–83.33	0.059	0.177
7	y = 4.62506e-005x - 0.174440	0.9997	0.83–83.33	0.382	1.09
8	y = 1.09862e-005x - 0.212514	0.9999	0.83–83.33	0.085	0.234
9	y = 3.95704e-005x -0.0973815	0.9999	0.83–83.3	0.278	0.625
10	y = 1.09242e-005x - 0.176053	0.9999	0.83–83.3	0.089	0.253
11	y = 1.01645e-005x - 0.173190	0.9998	0.83–83.3	0.120	0.296
12	y = 2.61995e-005x -0.0831525	0.9992	0.83–83.3	0.162	0.451

a) The calibration curves were constructed by plotting the peak areas versus the concentration of each analyte.

b) LOD refers to the limit of detection.

c) LOQ refers to the limit of quantification.

#### 3.3. Accuracy

The accuracy of the developed HPLC method was carried out by spiking the known amounts of analytes into extract solution of *P. pabularia*. After addition of known amounts of each analyte to the previously analysed extract solution recovery studies were examined. The results have been summed up in (Table 3). The recovery rates of the analytes ranged between 92.06 and108.02% with relative standard deviations in the range of 0.04–2.81% i.e., less than 5% indicating high accuracy of the developed method.

**Table 3:** Recovery percentage of marker compounds **1–12**.

Marker compounds	Amount present in the extract (ng)	Amount added (ng)	Amount found (ng)	Recovery (%)
1	2.95	4.3	6.913	92.16
2	2.70	4.3	6.897	95.74
3	10.83	4.3	15.121	99.79
4	2.03	4.3	6.039	92.06
5	18.82	4.3	22.83	93.25
6	81.79	4.3	85.76	92.32
7	113.09	4.3	117.29	97.67
8	3.53	4.3	7.449	91.13
9	8.05	4.3	12.695	108.02
10	4.93	4.3	9.325	102.20
11	11.93	4.3	16.075	96.39
12	18.233	4.3	22.821	106.76

#### 3.4. Precision

Based on the triplicate analysis carried out each day and also per day over a three day period, the intra as well as inter day precision levels for the developed method was analysed. The results are presented in (Table 4). The intraday variations were carried out by analyzing six replicates at three different concentrations (16.6, 41.6 and 83.3) in a day. The inter-day variations were determined by analyzing six replicates at three different concentrations over three days and the results are expressed as RSD(%)  =  (SD/mean) ×100%. The RSD was well below 5% indicating good precision of the developed method.

**Table 4:** Intra and inter-day precisions area of HPLC method of marker compounds **1–12**.

Marker compounds	Amount (µg/mL)	Intra-day precisions (n = 6)		Inter-day precisions (n = 6)	
		Mean area %RSD		Mean area %RSD	
1.	16.66	528127.5	1.19	499428.5	0.89
	41.66	1349015.66	0.59	1325150.8	2.54
	83.33	2624872.66	0.40	2552428.33	2.69
**2.**	16.66	369255	0.84	341051.5	2.48
	41.66	905746.33	0.70	869846	0.53
	83.33	1748411	0.32	1699871.5	0.30
**3.**	16.66	353819	2.62	488314.5	2.29
	41.66	1007683	0.616	1268067.5	0.35
	83.33	1948529.33	0.157	1995617	0.71
4.	16.66	2869202.05	0.29	2648853.6	1.40
	41.66	7436826.33	1.14	6703946.5	0.62
	83.33	14245153	0.48	13244343.5	0.54
5.	16.66	1721615.5	0.65	1640512	0.07
	41.66	4492011.66	0.48	4191352.5	0.76
	83.33	8696901	0.012	8325995.5	1.22
6.	16.66	2061524	0.32	2069826.5	0.88
	41.66	5472159	0.84	5190008.5	1.59
	83.33	10471469	0.02	9933193	0.57
7.	16.66	354990	0.09	361795	2.75
	41.66	975354.33	1.09	930367	0.55
	83.33	1888251.33	0.10	1800772	0.44
8.	16.66	1514109	0.39	1506029	0.35
	41.66	4058056.33	0.70	3911466	2.81
	83.33	7873578	0.35	7642937	0.92
9.	16.66	428799	1.83	419923	1.11
	41.66	1146641	0.24	1056793	1.03
	83.33	2224018.66	0.46	2113702.5	0.73
10.	16.66	1561757	0.50	1508290.5	0.14
	41.66	4144652.33	0.57	3907895.5	2.29
	83.33	8002167	0.26	7977745.5	0.73
11.	16.66	1665501	0.24	1600678	0.16
	41.66	4465353	0.96	4204301	1.75
	83.33	8569092	0.04	8282624.5	1.40
12	16.66	618911.5	1.01	588430.5	1.44
	41.66	1841182.66	1.69	1646506.5	0.32
	83.33	3281828	0.31	3178767	0.63

To test the repeatability of the method, the Relative standard deviation (RSD) values of retention times and peak area were determined and found to be <0.43% and 3.1% respectively.

#### 3.5. Percentage of different constituents in *P. pabularia*

The amount of isolated constituents present in DCM:methanol (1∶1) extract *P. pabularia* was found to be 0.123% (**1**), 0.150% (**2**), 0.460% (**3**), 0.050% (**4**), 0.801% (**5**), 0.368% (**6**), 0.623% (**7**), 0.022% (**8**), 0.325% (**9**), 0.101% (**10**), 0.455% (**11**) and 0.680% (**12**) on dry weight basis. Heraclenol (**5**) and osthol (**12**) the most active coumarin isolated from the root parts proved to be the most abundant constituents. The results of the quantification are summarised in (Table 5).

**Table 5:** Content of marker compounds **1–12** (%w/w) in *Prangos pabularia* determined by RP- HPLC.

Marker compounds	DCM: MeOH (1∶1) extract
**6-hydroxy-coumarin (1)**	0.123±0.001
**Umbelliferone (2)**	0.150±0.001
**Heraclenol glycoside(3)**	0.460±0.002
**Xanthotoxol (4)**	0.050±0.001
**Heraclenol (5)**	0.801±0.002
**Oxypeucedanin hydrate (6)**	0.368±0.003
**8-((3,3-dimethyloxiran-2-yl)methyl)-7-methoxy-2H-chromen-2-one (7)**	0.623±0.002
**Oxypeucedanin hydrate monoacetate (8)**	0.022±0.001
**Xanthotoxin (9)**	0.325±0.001
**4-((2-hydroxy-3-methylbut-3-en-1-yl)oxy)-7H-furo[3,2-g]chromen-7-one (10)**	0.101±0.001
**Imperatorin (11)**	0.455±0.002
**Osthol (12)**	0.680±0.003

## Conclusions

To the best of our knowledge this is the first study on the isolation, characterisation, bioevaluation, HPLC quantification and validation of chemical constituents of *P. pabularia*. The findings in this study demonstrate the high cytotoxic potential of this plant in an *in vitro* manner which is attributed mainly to the occurrence of osthol screened via MTT cytotoxicity assay. Further the presence of the highly bioactive compounds, Heraclenol (**5**) and osthol (**12**), in high concentrations (0.801%) and (0.680%) opens new avenues with regard to drug development. Validation results demonstrated that the developed method is simple, sensitive, selective and repeatable; it can be extended to evaluate the quality of *P. pabularia*. This first RP-HPLC fingerprint method can be helpful for the rapid analysis of its phytomolecules in various herbs/herbal formulation/plant products.

## Supporting Information

Data S1 **File contains Figures S1–S12.** Figure S1. Chromatograph and calibration curve of 6-hydroxo-couramin (1). Figure S2. Chromatograph and calibration curve of Umbelliferone (2). Figure S3. Chromatograph and calibration curve of Heraclenol glycoside (3). Figure S4. Chromatograph and calibration curve of Xanthotoxol (4). Figure S5. Chromatograph and calibration curve of Heraclenol (5). Figure S6. Chromatograph and calibration curve of Oxypeucedanin hydrate (6). Figure S7. Chromatograph and calibration curve of 8-((3, 3-dimethyloxiran-2-yl)methyl)-7-methoxy-2H-chromen-2-one (7). Figure S8. Chromatograph and calibration curve of Oxypeucedanin hydrate monoacetate (8). Figure S9. Chromatograph and calibration curve of Xanthotoxin (9). Figure S10. Chromatograph and calibration curve of 4-((2-hydroxy-3-methylbut-3-en-1-yl)oxy)-7H-furo[3,2-g]chromen-7-one (10). Figure S11. Chromatograph and calibration curve of Imperatorin (11). Figure S12. Chromatograph and calibration curve of Osthol (**12**).
(DOCX)
