# Correction: Isolation, Cytotoxicity Evaluation and HPLC-Quantification of the Chemical Constituents from *Prangos pabularia*

**DOI:** 10.1371/journal.pone.0115110

**Published:** 2014-12-03

**Authors:** The PLOS ONE Staff

The affiliation for the fifth author, Javid Ahmad Banday, is incorrect. Javid Ahmad Banday’s correct affiliation is “Department of Chemistry, National Institute of Technology, Srinagar 190006, India”.
