# Supplementary material for: Isolation, Cytotoxicity Evaluation and HPLC-Quantification of the Chemical Constituents from Prangos pabularia
Source: PLoS One. 2014 Oct 14;9(10):e108713. doi: 10.1371/journal.pone.0108713 (PMC4196845; doi:10.1371/journal.pone.0108713)
Supplement: Data S1 — File contains Figures S1–S12. Figure S1. Chromatograph and calibration curve of 6-hydroxo-couramin (1). Figure S2. Chromatograph and calibration curve of Umbelliferone (2). Figure S3. Chromatograph and calibration curve of Heraclenol glycoside (3). Figure S4. Chromatograph and calibration curve of Xanthotoxol (4). Figure S5. Chromatograph and calibration curve of Heraclenol (5). Figure S6. Chromatograph and calibration curve of Oxypeucedanin hydrate (6). Figure S7. Chromatograph and calibration curve of 8-((3, 3-dimethyloxiran-2-yl)methyl)-7-methoxy-2H-chromen-2-one (7). Figure S8. Chromatograph and calibration curve of Oxypeucedanin hydrate monoacetate (8). Figure S9. Chromatograph and calibration curve of Xanthotoxin (9). Figure S10. Chromatograph and calibration curve of 4-((2-hydroxy-3-methylbut-3-en-1-yl)oxy)-7H-furo[3,2-g]chromen-7-one (10). Figure S11. Chromatograph and calibration curve of Imperatorin (11). Figure S12. Chromatograph and calibration curve of Osthol (12). (DOCX) [file pone.0108713.s001.docx]

**Isolation, cytotoxicity evaluation and HPLC-quantification of the chemical constituents from *Prangos pabularia***

**Isolation, cytotoxicity evaluation and HPLC-quantification of the chemical constituents from *Prangos pabularia***

Saleem Farooq,^a^* Shakeel-u-Rehman,^b,c^ Nisar Ahmad Dangroo,^a^ Dev Priya,^d^ Javid Ahmad Banday,^c^ Pyare Lal Sangwan,^a^ Mushtaq Ahmad Qurishi,^c^ Surrinder Koul,^a^* and Ajit Kumar Saxena^d^

**^a^**Bio-organic Chemistry Section, CSIR-Indian Institute of Integrative Medicine, Canal road Jammu, India

**^b^**Bio-organic Chemistry Section, CSIR-Indian Institute of Integrative Medicine, Sanatnagar Srinagar, India

**^c^**Department of Chemistry, University of Kashmir, Hazratbal, Srinagar, India

*^d^Cancer Pharmacology Division, CSIR-Indian Institute of Integrative Medicine, Canal road Jammu, India*

**Figure S1.** Chromatograph and calibration curve of 6-hydroxo-couramin (**1**)

**Figure S2.** Chromatograph and calibration curve of Umbelliferone (**2**)

**Figure S3.** Chromatograph and calibration curve of Heraclenol glycoside (**3**)

**Figure S4.** Chromatograph and calibration curve of Xanthotoxol (**4**)

**Figure S5.** Chromatograph and calibration curve of Heraclenol (**5**)

**Figure S6.** Chromatograph and calibration curve of Oxypeucedanin hydrate (**6**)

**Figure S7.** Chromatograph and calibration curve of 8-((3, 3-dimethyloxiran-2-yl)methyl)-7-methoxy-2H-chromen-2-one (**7**)

**Figure S8.** Chromatograph and calibration curve of Oxypeucedanin hydrate monoacetate (**8**)

**Figure S9.** Chromatograph and calibration curve of Xanthotoxin (**9**)

**Figure S10.** Chromatograph and calibration curve of 4-((2-hydroxy-3-methylbut-3-en-1-yl)oxy)-7H-furo[3,2-g]chromen-7-one (**10**)

**Figure S11.** Chromatograph and calibration curve of Imperatorin (**11**)

**Figure S12.** Chromatograph and calibration curve of Osthol (**12**)
